# High accuracy in lower limb alignment analysis using convolutional neural networks, with improvements needed for joint‐level metrics

**DOI:** 10.1002/ksa.12481

**Published:** 2024-09-22

**Authors:** Christof Hoffmann, Fatih Göksu, Isabella Klöpfer‐Krämer, Julius Watrinet, Philipp Blum, Sven Hungerer, Steffen Schröter, Fabian Stuby, Peter Augat, Julian Fürmetz

**Affiliations:** ^1^ Department of Trauma Surgery BG Trauma Center Murnau Murnau Germany; ^2^ Department of Orthopedics and Reconstructive Surgery Diakonie Klinikum, GmbH Jung‐Stilling‐Krankenhaus Siegen Germany; ^3^ Institute for Biomechanics Paracelsus University Salzburg Salzburg Austria; ^4^ Department of Orthopaedic Sports Medicine Technical University Munich Germany; ^5^ Department of Orthopedics and Trauma Surgery, Musculoskeletal University Center Munich (MUM) University Hospital, LMU Munich Munich Germany

**Keywords:** deep learning convolutional neural network, leg deformity, long leg standing radiographs, osteotomy, total knee arthroplasty

## Abstract

**Purpose:**

Evaluation of long‐leg standing radiographs (LSR) is a standardised procedure for analysis of primary or secondary deformities of the lower limbs. Deep‐learning convolutional neural networks (CNN) offer the potential to enhance radiological measurement by increasing reproducibility and accuracy. This study aims to evaluate the measurement accuracy of an automated CNN‐based planning tool (mediCAD® 7.0; mediCAD Hectec GmbH) of lower limb deformities.

**Methods:**

In a retrospective single‐centre study, 164 pre‐ and postoperative bilateral LSRs with uni‐ or bilateral posttraumatic knee arthritis undergoing total knee arthroplasty (TKA) were enroled. Alignment parameters relevant to knee arthroplasty and deformity correction were analysed independently by two observers and a CNN. The intraclass correlation coefficient (ICC) was used to evaluate the accuracy between observers and the CNN, which was further evaluated using absolute deviations, limits of agreement (LoA) and root mean square error (RMSE).

**Results:**

CNN evaluation demonstrated high consistency in measuring leg length (ICC > 0.99) and overall lower limb alignment measures of mechanical tibio‐femoral angle (mTFA) (ICC > 0.97; RMSE < 1.1°). The mean absolute difference between angular measurements were low for overall lower limb alignment (mTFA 0.49–0.61°) and high for specific joint angles (aMPFA 3.86–4.50°). Accuracy at specific joint angles like the mechanical proximal tibial angle (MPTA) and the mechanical lateral distal femur angle (mLDFA) varied between lower limbs with deformity, with and without TKA with greatest difference for TKA (ICC 0.22–0.85; RMSE 1.72–3.65°).

**Conclusion:**

Excellent accuracy was observed between manual and automated measurements for overall alignment and leg length, but joint‐level metrics need further improvement especially in case of TKA similar to other existing algorithms. Despite the observed deviations, the time‐efficient nature of the algorithm improves the efficiency of the preoperative planning process.

**Level of Evidence:**

Level IV.

**Abbreviations**
aLDFA: anatomical lateral distal femur angle
AMA: anatomical mechanical angle
aMPFA: anatomical medial proximal femur angle
CNN: convolutional neural networks
ICC: intraclass correlation coefficient
JLCA: joint‐line convergence angle
LoA: limits of agreement
LSR: long‐leg standing radiographs
mLDFA: mechanical lateral distal femur angle
mLDTA: mechanical lateral distal tibia angle
mLPFA: mechanical lateral proximal femur angle
mMPTA: mechanical medial proximal tibia angle
mTFA: mechanical tibio‐femoral angle
OA: osteoarthritis
RMSE: root mean square error
SD: standard deviation
TKA: total knee arthroplasty

## INTRODUCTION

The evaluation of pre‐ and postoperative bilateral long‐leg standing radiographs (LSR) is a standardised method to asses deformities of the lower limb [1, 10, 25]. In clinical routine, LSRs are typically evaluated manually through landmark detection. However, anatomic alignment have shown considerable variability in intra‐ and interobserver reliability, ranging from poor to excellent [3, 4, 6, 11, 20, 24]. Reliable measurements independent of the examiner are relevant both for correct treatment decisions and for the scientific evaluation of the results of total knee arthroplasty (TKA) and osteotomies.

Computer‐assisted landmark setting and analysis, using deep‐learning convolutional neural networks (CNN), have previously been shown to enhance intraobserver reliability and reduce the time required for analysis [9, 26]. CNN assisted measurement of leg length and deformity has been identified to improve medical care by providing greater accuracy while saving both time and resources [15, 16, 17, 21, 22, 29]. Recent studies indicate that CNN match or even surpass human performance in assessment of LSRs [24]. However, these previously tested commercially available models do not offer the additional planning tools for TKA or osteotomies, which are needed in daily clinical practice.

The aim of this study was to assess the performance of a CNN implemented in a common planning software (mediCAD® 7.0; mediCAD Hectec GmbH) that allows analysis and preoperative planning. It was hypothesized that CNN measurements could serve as a reliable alternative to manual measurements including patients with deformities and TKA.

## MATERIALS AND METHODS

This retrospective study was conducted in accordance with local data protection regulations, and institutional Review Board approval was obtained (Reference No. 20‐0859).

All LSRs were performed in 2020 for preoperative planning and postoperative control within the first 6 weeks following surgery and anonymised before inclusion to the study. Inclusion criteria consisted of adults aged 18 years or older who had undergone or were planned for TKA implantation due to posttraumatic knee osteoarthritis (OA). Exclusion criteria included image artifacts, nonstandardized limb positioning during imaging, joint‐line overlay caused by extension deficits, abnormal stitching, missing calibration balls or arthrodesis of the knee or ankle joint. A total of 180 bilateral weight‐bearing LSRs from 90 consecutive patients were screened for inclusion. The a priori power analysis, with a power of 80% and *α* = 0.05, indicated that a correlation of *r* ≥ 0.3 can be detected with a minimum sample size of 84 cases. Eight patients had to be excluded from the study for following reasons: 2x unilateral knee arthrodesis, 4x joint‐line overlay, 1x missing calibration ball, 1x artifact. As a result, a total of 164 weight bearing LSRs (affected and unaffected limb) preoperatively, and 164 weight‐bearing bilateral LSRs (affected and unaffected limb) postoperatively from 82 patients who were included in this study.

All LSRs were acquired from the same device (Digital Diagnost 4.1.9 X‐Ray‐System PhillipsHealthcare) and included a calibration marker (ball, 25 mm diameter) placed either medial or lateral to the knee joint. Standardised images were obtained with the patient standing on a weight‐bearing platform under compensation of leg length discrepancy and the patellae‐oriented anteriorly.

The following parameters were manually and automatically identified:

- mechanical tibio‐femoral angle [mTFA]
- anatomical mechanical angle (AMA)
- leg length
- mechanical lateral proximal femur angle (mLPFA)
- joint‐line convergence angle (JLCA)
- mechanical lateral distal femur angle (mLDFA)
- mechanical medial proximal tibia angle (mMPTA)
- mechanical lateral distal tibia angle (mLDTA)
- anatomical medial proximal femur angle (aMPFA)
- anatomical lateral distal femur angle (aLDFA)

### Manual measurements

Manual measurements were performed according to the mediCAD® software recommendations (mediCAD® 7.0; Modul 3D Knee version 2.5.77.15961; mediCAD Hectec GmbH), following the guidelines of Paley [19]. Two experienced orthopaedic surgeons (C. H. and F. G.) evaluated the radiographs and were blinded to the CNN results. During manual measurements, detection of the calibration sphere was done automatically by the system and manually corrected if needed. The total time for both manual and automated limb analysis was measured.

### Automated measurements by CNN

**Figure 1:**
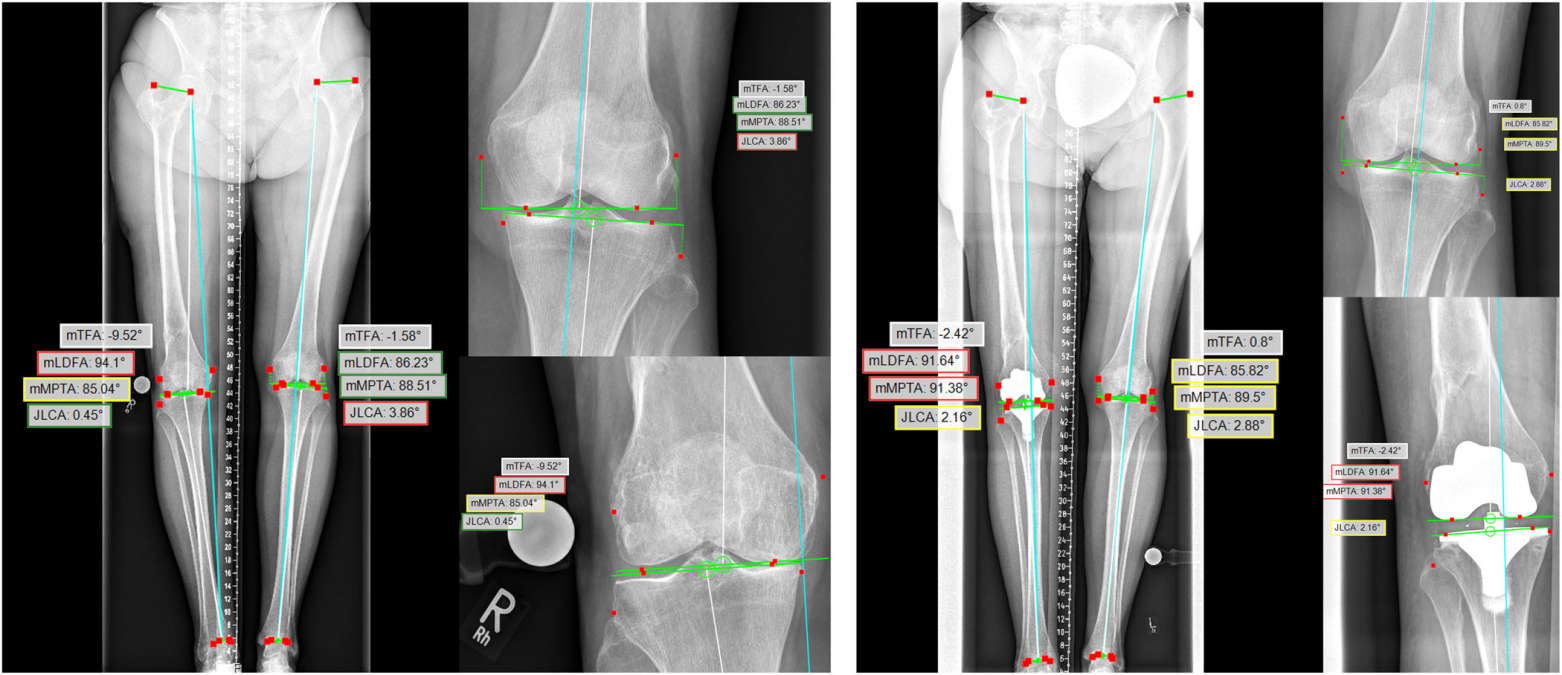
Automated CNN‐based measurements postoperatively after unilateral total knee arthroplasty. Landmarks close to the knee joint demonstrate deviations to the software recommendations. JLCA, joint‐line convergence angle; mLDFA, mechanical lateral distal femur angle; mMPTA, mechanical medial proximal tibia angle; mTFA, measurements are displayed here for mechanical tibio‐femoral angle.

The software for the analysis of LSRs is based on a CNN which was trained using radiographs from the Mulitcenter Osteoarthritis Study (MOST), Cohort Hip and Cohort Knee Study (CHECK) and Osteoarthritis Initiative Study (OAI) [7, 28]. All LSRs underwent standardised analysis using the mediCAD® software. Both observers initiated the automated software algorithm independently following their manual measurements (Figure 1).

### Statistics

To evaluate the diagnostic performance of the CNN‐based and observer‐based image analysis, intraclass correlation coefficients (ICC) between observer measurements and CNN measurements were calculated using IBM SPSS Statistics 26 (SPSS Inc.). All analyses were conducted separately for the affected leg ‘pre‐OA’ and ‘post‐TKA’, as well as for the ‘unaffected’ leg.

First, the inter‐rater reliability of observer 1 (C. H.) vs. observer 2 (F. G.) was assessed by ICC (two‐way mixed, single measures, absolute agreement) and averaged to establish a ‘ground truth’ [23]. Similarly, CNN data of both observers was averaged and the ICC (two‐way mixed, average measures, absolute agreement) was calculated for mean measurements of observer 1 + 2 (mean_observer_) vs. mean measurements of CNN (mean_CNN_) and interpreted according to Koo and Li [14]. Differences in ICC values between the different conditions (unaffected, before OA and after TKA) were tested using the Wilcoxon test. Significance level was set at *α* = 0.05.

The correspondence of manual and automatic landmark detection/measurements was evaluated using Scatter and Bland–Altman plots [2]. The limits of agreement (LoA) were determined based on errors deemed clinically relevant, following criteria from Knutson beeing >2° for angles and >5 mm for distances [13].

In addition, root mean square errors (RMSE) were calculated for deviations between mean _observer_ and mean _CNN_ for the most relevant parameters mTFA, mLDFA, and mMPTA. The time required for measurements was compared using student's *t* test.

## RESULTS

The median age of the patients was 62 years (range 43–89) with 57 male and 25 female patients.

Inter‐rater reliability between observer 1 and observer 2 showed good (ICC > 0.75) to excellent correlation (ICC > 0.9), while measurements of JLCA showed moderate reliability (ICC > 0.5).

The ICC values of *mean_observer_ versus mean_CNN_* are displayed in Table 1. A comparison of the ICCs across the conditions (unaffected, pre‐OA, post‐TKA) revealed significantly poorer performance of the CNN‐based algorithm in the presence of an implant (post‐TKA) (overall ICCs unaffected vs. post‐TKA: *p* = 0.016; overall ICCs unaffected vs. pre‐OA: *p* = 0.594).

**Table 1:** ICC values of mean_observer_ vs. mean_CNN_, green = excellent reliability, yellow = good reliability, orange = moderate reliability, red = low reliability.

	pre‐OA	post‐TKA	Unaffected
mTFA	1.00	0.98	0.99
AMA	0.82	0.89	0.86
Leg length	0.99	1.00	1.00
mLPFA	0.78	0.55	0.64
JLCA	0.88	0.11	0.51
mLDFA	0.81	0.63	0.90
mMPTA	0.89	0.34	0.82
mLDTA	0.54	0.66	0.78
aMPFA	0.81	0.66	0.67
aLDFA	0.85	0.60	0.92

Abbreviations: aLDFA, anatomical lateral distal femur angle; AMA, anatomical mechanical angle; aMPFA, anatomical medial proximal femur angle; JLCA, joint‐line convergence angle; mLDFA, mechanical lateral distal femur angle; mLDTA, mechanical lateral distal tibia angle; mLPFA, mechanical lateral proximal femur angle; mMPTA, mechanical medial proximal tibia angle; mTFA, mechanical tibio‐femoral angle; post‐TKA, postoperative following total knee arthroplasty; pre‐OA, preoperative with osteoarthritis; unaffected, unaffected contralateral side.

The mean absolute deviation of all angles was 1.1°. The mean absolute differences and the outliers are shown in Table 2 and the RMSE in Table 3.

**Table 2:** Absolute deviations of mean_observer_ and mean_CNN_ [°], [mm], and number of outliers >2° or >5 mm.

	Difference *M*	SD	Outliers ( >2° or >5 mm)
mTFA pre‐OA (°)	0.61	0.84	6
mTFA post‐TKA (°)	0.54	0.64	2
mTFA unaffected (°)	0.49	0.36	0
AMA pre‐OA (°)	0.69	0.66	3
AMA post‐TKA (°)	0.57	0.50	2
AMA unaffected (°)	0.60	0.42	0
Leg length pre‐OA (mm)	4.65	7.5	19
Leg length post‐OA (mm)	3.42	3.96	14
Leg length unaffected (mm)	3.73	7.35	12
mLPFA pre‐OA (°)	3.81	5.14	42
mLPFA post‐TKA (°)	4.76	10.0	43
mLPFA unaffected (°)	3.95	7.37	41
mLDFA pre‐OA (°)	2.03	2.10	34
mLDFA post‐TKA (°)	1.34	2.74	11
mLDFA unaffected (°)	1.22	1.21	13
mMPTA pre‐OA (°)	2.43	2.23	41
mMPTA post‐TKA (°)	1.77	3.20	20
mMPTA unaffected (°)	1.77	1.54	32
JLCA pre‐OA (°)	2.44	2.01	42
JLCA post‐TKA (°)	1.26	1.98	12
JLCA unaffected (°)	1.42	1.26	21
mLDTA pre‐OA (°)	4.26	5.97	50
mLDTA post‐TKA (°)	3.91	3.56	55
mLDTA unaffected (°)	3.38	2.58	50
aMPFA pre‐OA (°)	3.86	5.20	41
aMPFA post‐TKA (°)	4.50	8.42	42
aMPFA unaffected (°)	4.04	7.43	43
aLDFA pre‐OA (°)	1.98	2.07	30
aLDFA post‐TKA (°)	1.67	3.59	12
aLDFA unaffected (°)	1.21	1.19	13

Abbreviations: aLDFA, anatomical lateral distal femur angle; AMA, anatomical mechanical angle; aMPFA, anatomical medial proximal femur angle; JLCA, joint‐line convergence angle; mLDFA, mechanical lateral distal femur angle; mLDTA, mechanical lateral distal tibia angle; mLPFA, mechanical lateral proximal femur angle; mMPTA, mechanical medial proximal tibia angle; mTFA, mechanical tibio‐femoral angle; post‐TKA, postoperative following total knee arthroplasty; pre‐OA, preoperative with osteoarthritis; unaffected, unaffected contralateral side.

**Table 3:** RMSE values [°] between mean_observer_ vs. mean_AI_.

	pre‐OA	post‐TKA	Unaffected
mTFA	1.0	0.8	0.6
mLDFA	2.9	3.0	1.7
mMPTA	3.3	3.7	2.3

**Figure 2:**
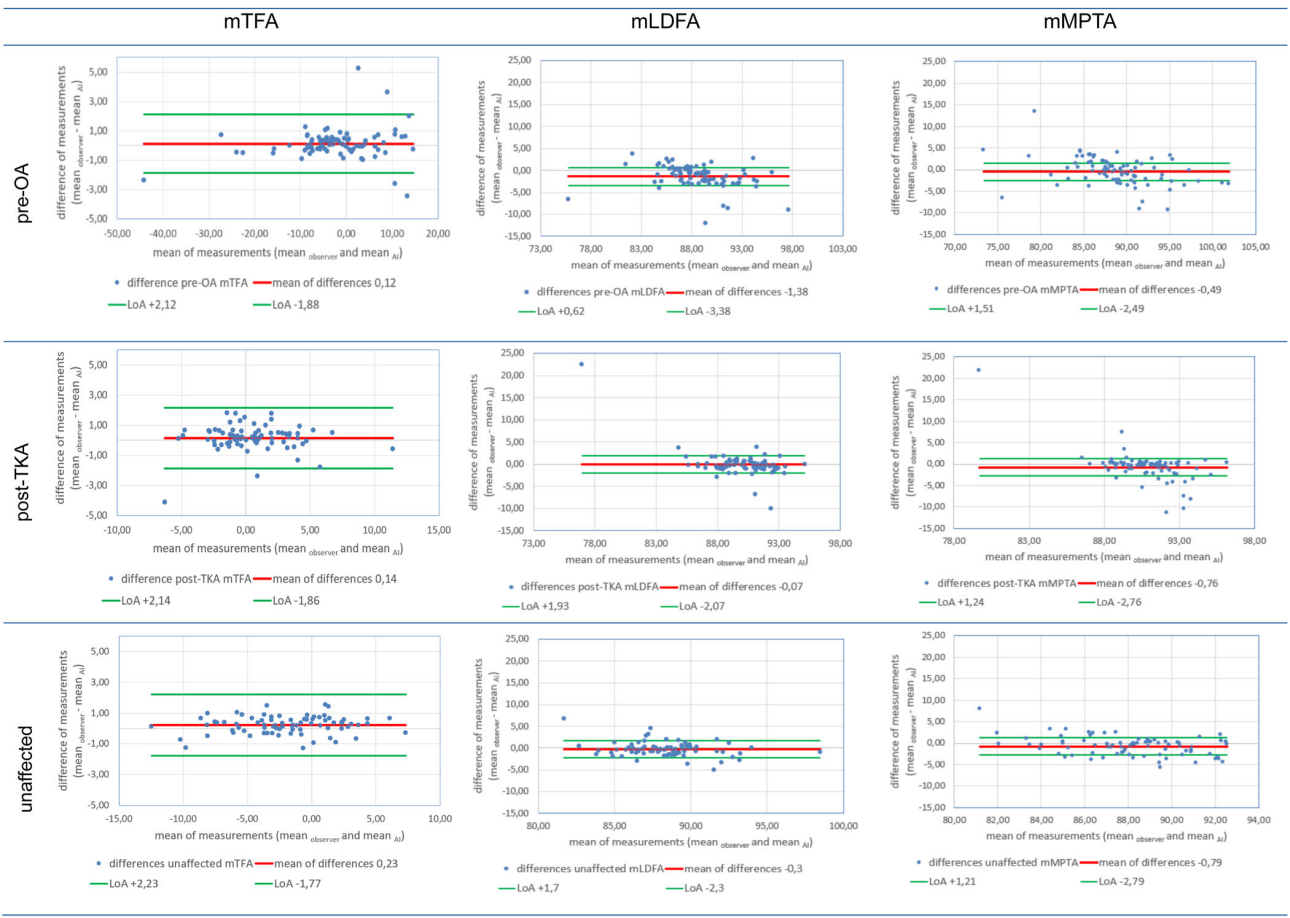
Bland–Altman plots of manual versus automatic measurement of mTFA, mLDFA, and mMPTA including all LSRs (OA preoperatively [pre‐OA] and TKA postoperatively [post‐TKA] and unaffected knees [unaffected]). The limits of agreement (LoA) are defined within a range of ±degrees (green line) from the mean difference between manual and automatic measurement (red line).

The agreement between manual and automatic measurements is illustrated by plotting the differences between two measurements against their average (Figure 2). The absence of systematic biases is indicated by mean lines closely clustered around zero, with no discernible trends observed as mean values change. The higher deviations observed for joint angles, such as mLDFA and mMPTA, were primarily due to outliers, as the majority of measurements fell within ±2° from the mean of measurements.

The automated CNN‐based analysis required significantly less time (16 s) compared to the manual analysis (85 s; *p* < 0.001).

Inter‐rater ICC coefficients for CNN of both observers showed excellent to moderate reliability (*r* = 0.78–1.00).

## DISCUSSION

The most important finding of this study was that the automated CNN‐based analysis demonstrated excellent accuracy for overall lower limb alignment and leg length, but showed significant deviations in joint‐level measurements, particularly in cases involving TKA.

These findings align with previous studies of CNN‐based algorithms, where deviations of joint angle measurements have been higher compared to leg length or overall alignment measurements [12, 15, 16, 17, 22, 23, 24, 27, 29].

Higher deviations in the joint angles can be attributed to the proximity of the landmarks, where even small deviations can have major effects on the angle measurement. The mediCAD® CNN algorithm was mainly trained with images without implants, which likely reduces accuracy in the presence of a knee implant. In addition, postoperative knee extension deficit might also influence accuracy [5].

Interestingly, the CNN‐based algorithm did not lead to identical angle measurements when initiated by the two investigators. This discrepancy was particularly evident in the mLDFA.

In previous studies, a different software algorithm (LAMA; IB Lab GmbH) for automated analysis of LSR was evaluated. Similar to this study, results were best in regards to leg length and mTFA [23, 24, 27]. The performance of the algorithm demonstrated lower absolute deviations compared to the algorithm evaluated in this study, as indicated by higher ICC values both with and without TKA [23, 24, 27]. The superior performance of the LAMA algorithm is likely due to its training with a large data set, comprising more than 15,000 images [24]. Nevertheless, clinically relevant deviations of more than 2° in the joint angles around the knee have also been observed for the LAMA algorithm in a relevant number of up to 37% of cases, which poses the need for manual readjustment [18]. Another algorithm focusing only on mTFA and AMA has indicated very high ICC values (>0.99) in a wide range of morphological configurations while indicating a need for improvement in the measurement of joint‐level metrics [22].

No details of the ICC model used for the calculation were provided in the aforementioned studies, which limits comparability [18, 22, 23, 24, 27]. Exploring landmark detection failures in CNN algorithms and incorporating patient‐specific factors like BMI and height could enhance the algorithm's robustness.

### Limitations

The reasons for deviations in landmark detection by the CNN algorithm remain somewhat unclear, largely due to the inherent ‘black box’ nature of artificial intelligence algorithms [8]. The investigated cohort was characterised by severe posttraumatic osteoarthritis, which may limit generalisability, although analysis of the contralateral side provides insights into performance with less deformity and osteoarthritis. Unilateral LSR were excluded from the study, limiting data diversity.

## CONCLUSIONS

The CNN algorithm demonstrated excellent accuracy for measuring overall alignment and leg length, but improvements are needed for joint‐level measurements and TKA cases. Despite the observed deviations, the time‐efficient nature of the algorithm improves the efficiency of the preoperative planning process.

## AUTHOR CONTRIBUTIONS

Julian Fürmetz, Steffen Schröter and Peter Augat conceived and planned the experiments. Christof Hoffmann and Fatih Göksu carried out the manual measurements. Isabella Klöpfer‐Krämer was responsible for data collection and analysis. Steffen Schröter, Peter Augat, Isabella Klöpfer‐Krämer and Julian Fürmetz contributed to the interpretation of the results. Julian Fürmetz took the lead in writing the manuscript. All authors provided critical feedback and helped shape the research, analysis and manuscript.

## CONFLICT OF INTEREST STATEMENT

Steffen Schröter and Julian Fürmetz were expert members of the AO deformity correction planning task force collaborating with mediCAD®. The remaining authors declare no conflict of interest.

## ETHICS STATEMENT

The study was approved by the local ethics committee (Reference No. 20‐0859, Munich). All patient information from radiographic images was deidentified and patient consent was not required.

## Data Availability

The data that support the findings of this study are available on request from the corresponding author (J. F.).
